# Vitamin D abnormalities of children with recurrence of malignancy and comparison with newly diagnosed patients

**DOI:** 10.22088/cjim.13.4.735

**Published:** 2022

**Authors:** Nahid Reisi, Azar Mirzaei, Alireza Moafi, Pouran Raeissi, Maryam Naghdhassani

**Affiliations:** 1 Department of Pediatric Hematology and Oncology, Faculty of Medicine, Child Growth and Development Research Center and Isfahan Immunodeficiency Research Center, Isfahan University of Medical Sciences, Isfahan, Iran; 2 Department of Pediatrics, Isfahan University of Medical Sciences, Isfahan, Iran; 3 Department of Health Service Management, School of Health Management and Medical Information Science, Iran University of Medical Sciences, Tehran, Iran; 4 Department of Psychology, Azad University, Najaf Abad Branch, Isfahan, Iran

**Keywords:** Vitamin D, Recurrence, Cancer, Children

## Abstract

**Background:**

Vitamin D (Vit-D) is a necessary ingredient for human growth and its deficiency may increase the risk of cancer and its recurrence. The main purpose of this research was to assess the levels of Vit-D in children with recurrence of malignancy and compare it with new cases of malignancy and the control group.

**Methods:**

The status of 25(OH) Vit-D was determined utilizing the HPLC method in 47 patients with recurrence of malignancy (group A), 50 children with new malignancy (group B) and 49 normal healthy siblings of the two groups as a control (group C).

**Results:**

Vit-D was low (<30 ng/ml) in the 92% of patients with recurrence of malignancy, which was a significant difference compared to groups B (60%) and C (45%). Vit-D insufficiency (10-30 ng/dl) in group A was also higher than the other two groups. The mean levels of Vit-D in patients with recurrence were significantly lower than the new cases and controls. Low Vit-D (<30 ng/ml) in group A in both male and female, and also in all ages (<6 and ≥ 6 years) was higher than groups B and C. Also, low Vit-D in terms of the type of malignancy in group A was higher than group B only in leukemic patients while this was not different for non-leukemic patients in these two groups.

**Conclusion:**

Results of this study showed an increased prevalence of low Vit-D in children with recurrence of malignancies. Therefore, it may increase the risk of recurrence of malignancies in children.

Vitamin D is now recognized as a precursor to calcitriol (1, 25-dihydroxyVitamin D), an essential ingredient for calcium homeostasis and bone growth. In addition to skeletal activity, Vit-D has many extra-skeletal activities that are essential for the normal functioning of the immune, metabolic, respiratory, reproductive and cutaneous systems (1-4). Deficiency of this vitamin not only leads to skeletal abnormalities but also can increase the risk of autoimmune, chronic inflammatory and malignant diseases (3, 5, 6). The main source of Vit-D (90%) in humans is from sun exposure. UV-B radiation of the sunlight converts skin substrate 7dehydrocholesterol into cholecalciferol (vitamin D3). The rest (10%) is from the daily intake of food in two shapes, ergocalciferol (Vit- D2) or cholecalciferol (Vit- D3). Following food absorption, Vit- D2 or Vit- D3 is converted to 25-hydroxyVitamin D2 or D3 in the liver and next hydroxylated by the cytochrome P450 enzyme CYP27B1 to shape 1, 25-dihydroxyvitamin D (calcitriol) in the kidneys, placenta, and other tissues (7-9). 1, 25-dihydroxyvitamin D controls about 5% of the human genome by binding to and activating the nuclear Vit-D receptor (VDR) that is present in most body cells, such as the brain, heart, gut, skin, gonads and activated lymphocytes. It can prevent the development of numerous diseases such as cancer by regulating the signaling pathways involved in cell proliferation, differentiation and growth arrest.

A wide variety of body tissues including breast, ovary, uterus, prostate, pancreas, colon and skin have extrarenal CYP27B1-mediated production of 1, 25-dihydroxyvitamin D that is effective for the regulation of cell proliferation and cancer prevention in these tissues (2, 3, 10). (2, 10-13). Low Vit-D status can be in relation to higher incidence of cancer and poorer prognosis (9, 14-18). Today, most studies in this area have been done in adult malignancies (8, 9, 18, 19) and the number of these studies in children are lower than in adults. Furthermore, we cannot find any study in this regard in children with recurrence of malignancies. The main goal of this research was to evaluate the status of Vit- D in children with recurrence of malignancies and compare it with Vit- D levels in new cases of malignancy and the control group.

## Methods

**Study design and population:** In a case-control study from June 2018 to July 2019, 25(OH) Vit-D levels were measured and compared in 3 groups, including 47 subjects with recurrence of previous malignancy (group A), 50 children with newly diagnosed malignancy (group B) and 49 normal healthy siblings of the two groups as a control group (group C) in the Children's Cancer Center of Isfahan University of Medical Sciences. In addition, the correlation of Vit-D levels and demographic characteristics of the studied patients (age, sex and type of malignancy) was also investigated. 

The sample size for each group was statistically calculated to be 50 children using the following equation:

$n=\frac{{\left(Z_{1-\frac{\propto }{2}}+Z_{1-\beta }\right)}^{2}({\mathrm{SD}}_{1}^{2}+{\mathrm{SD}}_{2}^{2})}{d^{2}}=\frac{{\left(1.96+0.84\right)}^{2}(10.8^{2}+6.9^{2})}{5.3^{2}}\approx 50$

The aim of the research was described to the patients' parents and informed consent was acquired from parents who agreed with their child's participation in the study. Qualified cases were chosen according to the inclusion and exclusion criteria. 

**Inclusion and exclusion criteria:** Age less than 18 years and the diagnosis of malignancy or recurrence of it based on bone marrow examination and flow cytometry method for hematologic malignancies (ALL/AML) and pathology studies and immunohistochemistry (IHC) staining for non-hematologic cases were inclusion criteria (20). The presence of illnesses such as hypoparathyroidism, gastrointestinal, liver and kidney diseases, Down syndrome, obesity and short stature, or conditions such as Vit-D intake for at least the past three months and taking medications such as anticonvulsants that affect Vit-D metabolism were considered as exclusion criteria (21).

**Data collection**: Demographic data of the subjects, including age, sex and type of malignancy (ALL/AML or non- ALL/AML) were recorded in a special form. The age was recorded in years and categorized into two groups, those below six years and those above six years (18). A venous blood sample measuring at least 3 cc was taken of all participants in the first admission to hospital and sent to the laboratory within one hour of collection of the sample for determination of 25(OH) Vit-D levels (18).

**Laboratory measurements: **The serum levels of 25(OH) Vit-D were measured by the high-performance liquid chromatography (HPLC) method (Agilent, USA) and were classified into four levels as follows:

- < 10 ng/ml (Deficient)
- 10-30 ng/ml (Insufficient)
- 30-100 ng/ml (Normal)
- > 100 ng/ml (Toxic)

The cut-off value to define low vitamin D was Vit-D level less than 30 ng/ml (18). 

**Ethics Statement**: The Research and Ethics Committees of Isfahan University of Medical Sciences approved this study (Code: 396801and IR.MUI.REC.1396.3.801, respectively). 

**Data Analysis: **The collected data were analyzed with SPSS Version 21.0 (Released 2012. Armonk, NY: IBM Corp) and chi-square, Kruskal-Wallis and Mann-Whitney U tests at 5% significance level. The One-way ANOVA test was utilized to analyze the statistical difference between the mean of Vit-D in the study groups and the ANCOVA test was employed to adjust the effect of age (as an intervening variable). 

## Results

The 25(OH) Vit-D serum levels of 47 children with recurrence of malignancy, 50 children with new malignancy and 49 healthy children were assessed and compared using the method described above. The demographic characteristics of the three groups and the mean±SD of 25(OH) Vit-D serum of groups are shown in table 1. As can be seen, the mean age of the three groups was significantly different (P=0.02) and the mean age of group B was lower than groups A and C (P= 0.01, P= 0.01 respectively). Most of the patients were males and had leukemia (ALL/AML), but there was no significant difference in terms of sex and type of malignancy between groups A and B. Mean Vit-D levels were different in the three groups and this difference was also significant with age adjustment (p<0.001). Children with recurrence of malignancy had lower mean Vit-D levels than the newly diagnosed children and healthy control groups (P=0.01, p<0.001 respectively) but no significant difference between groups B and C (P= 0.14) was observed. 

**Figure 1:**
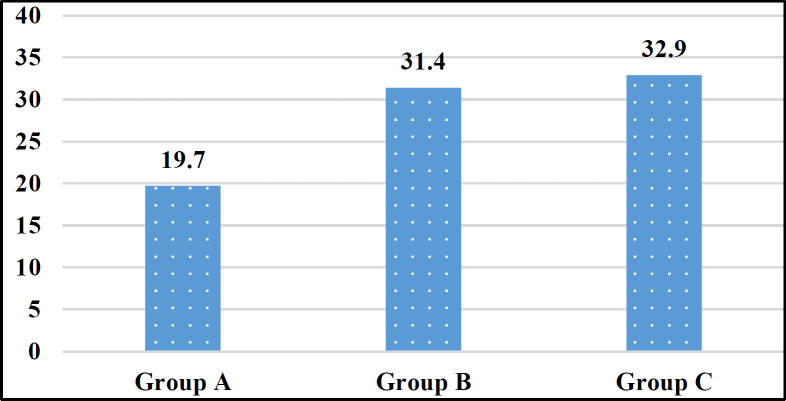
Mean levels of 25(OH) Vit-D in study groups (ng/ml)

The comparison of mean Vit-D levels in new and recurrence cancer patients versus healthy subjects and the distribution of Vit-D levels in study groups is shown in figure 1 and table 2, respectively.

**Table 1:** Demographic data of the three groups A, B, C and their mean ± SD of 25(OH) Vit-D levels

**Data**	**Group A** **(N=47)**	**Group B** **(N=50)**	**Group C** **(N=49)**	**P-value**
**Age (year)**				
Mean ± SD Range ≤ 6 > 6	7.6±3.7 3-17 24 23	5.8± 3.1 1-13 32 18	7.5± 3.8 2-15 25 24	0.02
**Sex**				
Male Female	28 19	26 24	29 20	0.65
**Type of malignancy**				
ALL/AML* Non-ALL/AML	39 8	40 10	- -	0.34
25(OH) Vit-D (ng/dl) Mean± SD	19.7± 11	31.4± 21.8	32.9± 16.1	<0.001

*ALL: Acute lymphoblastic leukemia, AML: Acute myeloblastic leukemia

**Table 2:** Distribution of Vit-D levels in groups A, B and C

Vitamin D		**Groups**			**P-value**
		**A**	**B**	**C**	
Deficient	Number	3	3	3	<0.001
	% Within group	6.4%	6.0%	6.1%	
Insufficient	Number	40	27	19	
	% within group	85.1%	54.0%	38.8%	
Normal	Number	4	20	27	
	% within group	8.5%	40.0%	55.1%	

In our study, generally 92% (n=43) of patients with recurrence of malignancy, 60% (n=30) of new patients and 45% (n=22) of healthy controls had low Vit-D levels (<30 ng/ml). Vit-D insufficiency was seen in 85.1% of children with recurrence of malignancy (group A), which was significantly higher than the new malignancy and control groups. This was also higher in new patients than in the control group (table 2). The study results also showed that when we evaluated cases with low Vit-D levels (< 30 ng/ml) based on sex, age and type of malignancy, Vit-D level < 30 ng/ml in the group with recurrence of malignancy in both male and female, and also in all ages (≤ 6 and > 6 years) was higher than the other two groups. Also, low Vit-D in terms of the type of malignancy was higher in group A than group B only in leukemic patients, while this was not different for non-leukemic patients in these two groups. Furthermore, the prevalence of low Vit-D in newly diagnosed children with age ≥6 was more than the control group (table 3). 

It was also found that the frequency of Vit-D abnormality was not significantly different for male and female and age ≤6 or ˃6 years in both group A and B (P= 0.87, P= 0.73 and P=0.18, P=0.63 respectively) but in the control group C, Vit-D abnormality was higher in children with age >6 years (P=0.001).

**Table 3:** Distribution of low 25(OH) Vit-D by sex, age and type of malignancy in the study groups

**P-value**	**Group C** **(N =49)**		**Group B ** **(N =50)**		**Group A** **(N=47)**		
0.004 0.01	48.3 40.0	14 8	57.7 62.5	15 15	8/92 89.5	26 17	**Sex** Male Female
0.001 0.02	20.0 70.8	5 17	62.5 55.6	20 10	79.2 92.3	19 24	**Age (year)** ≤6 >6
0.005 0.38	- -	- -	58.1 71.4	25 5	92.3 87.5	36 7	**Type of malignancy** ALL/AML Non-ALL/AML

Level < 30 ng/ml

## Discussion

The findings showed that children with malignancy, especially those with recurrence, had lower Vit-D levels than the control group and the prevalence of Vit-D insufficiency in patients with recurrence was higher than the new cases of malignancy and the control group. Children with cancer due to inadequate sun exposure, nutritional deficiencies, chemotherapy and radiation are susceptible to Vit-D deficiency (8, 22, 23). Also, the mean level of Vit-D in this group is usually lower than the children without cancer (24). Revuelta et al. in a meta-analysis showed that 14% (range 0-61.5%) of children with cancer were Vit-D deficient and 23% (range 0-83%) were insufficient (19). Mohan et al., also found that the incidence of Vit-D insufficiency (<30) in cancer children (80.59%) was higher than in controls (50.98%) (18). This deficiency is not only seen in children with malignancy but also in survivors of malignancies. Sinha et al. in a cross-sectional study found out that Vit-D deficiency (<10 ng/ml) in children with a history of malignancy was more common than the control group (21.3% vs. 3.3%, P=0.013) and also, the 25(OH) Vit-D levels in childhood cancer survivors were lower than the control group. (23). 

In another study carried out in all survivors treated with chemotherapy or chemotherapy plus cranial radiation, Reisi et al. found that Vit-D deficiency and insufficiency were greater in leukemia survivors than in their healthy siblings as a control. In this study, the mean levels of Vit-D in survivors were also lower compared to the control group (22). Nowadays, the effect of Vit-D in reducing the development, progression and recurrence of adult malignancies such as breast, colorectal and prostate has been identified, but there are a few studies about this in children (25-31). On the other hand, the majority of the research on children has determined Vit-D abnormalities in new cases and in survivors of malignancies, and few studies have been performed on recurrences of childhood malignancies. According to the results of this study, it may be possible to express the role of Vit-D in preventing the recurrence of childhood malignancies. The findings of this study indicated that there was no relationship between gender and low Vit-D in children with recurrence of malignancy and newly diagnosed cases. Similar results were obtained in the study of Reisi et al. and Mohan et al. in survivors of malignancies and Helou et al. in new cases of malignancy (8, 18, 22). Choudhary et al. in a retrospective chart review on 484 cancer survivors (234 males) have also concluded of no significant correlation between Vit-D insufficiency and gender (14). However, Culic et al. have found lower levels of Vit-D in newly diagnosed girls (32).

In our study, it was also found that the status of low Vit-D levels was no different at younger or older than 6 years in children with recurrence of malignancy and new cases. Revuelta et al. provided an electronic database about the relation between Vit-D level and cancer through no restricted searching and their results were similar to our findings (19). Our findings were also in concordance with findings achieved by Helou et al., Mohan et al. and Sinha et al. (8, 18, 23). 

In the present study, the high prevalence of low levels of Vit-D was seen in patients with leukemia recurrence, which was significantly different from new cases of the disease. Previous studies in new cases of malignancies have reported different results regarding the type of malignancy and low Vit-D levels. Helou et al.’s study showed that there was no association between type of cancer (leukemia and solid tumors) and Vit-D levels (8). Mohan et al. reported that it is more in leukemic patients (18). Revuelta et al. and Culic et al. showed that the prevalence of Vit-D abnormality is higher in new patients with solid malignant tumors (19, 20). Recent research has shown the association of low Vit-D status with a poorer prognosis in leukemia and lymphomas. Vit-D receptor (VDR) agonists can inhibit proliferation, induce differentiation, aggravate apoptosis and decrease pro-inflammatory cytokines production in hematologic malignancies and also have a synergistic effect with some chemotherapeutic drugs (22, 33). According to the results of this research, it seems that low Vit-D status may have an effect on the recurrence of childhood malignancies. 

The results of this study regarding the type of malignancy and low levels of Vit-D cannot be cited due to the small number of patients with non-leukemic malignancy and larger studies are suggested. The main limitations of this study were the impossibility to determine the exact status of the diet, the level of sun exposure and the amount of physical activity of the study groups, as well as the small sample size. Based on our results, it concluded that children with recurrence of malignancy, especially leukemia, have lower levels of Vit-D than in newly diagnosed cases of the disease and the control group and the prevalence of low Vit-D in this group is higher than in new cases of the disease. Therefore, low Vit-D levels may play a role in increasing the risk of recurrence of childhood malignancies.
